# SEM3De: image restoration for FIB-SEM

**DOI:** 10.1093/bioadv/vbad119

**Published:** 2023-09-06

**Authors:** Rayane Hamdane Serir, Aurelie Deliot, Caroline Kizilyaprak, Jean Daraspe, Christine Walczak, Françoise Canini, Amandine Leleu, Sergio Marco, Frederic Ronzon, Cedric Messaoudi

**Affiliations:** Multimodal Imaging Center, CNRS UAR2016, INSERM US43, Institut Curie, PSL Research University, Université Paris-Saclay, 91401 Orsay Cedex, France; Sanofi, 69280 Marcy-l'Étoile, France; Electron Microscopy Facility, University of Lausanne, 1015 Lausanne, Switzerland; Electron Microscopy Facility, University of Lausanne, 1015 Lausanne, Switzerland; Multimodal Imaging Center, CNRS UAR2016, INSERM US43, Institut Curie, PSL Research University, Université Paris-Saclay, 91401 Orsay Cedex, France; Sanofi, 69280 Marcy-l'Étoile, France; Sanofi, 69280 Marcy-l'Étoile, France; Sanofi, 69280 Marcy-l'Étoile, France; Sanofi, 69280 Marcy-l'Étoile, France; Multimodal Imaging Center, CNRS UAR2016, INSERM US43, Institut Curie, PSL Research University, Université Paris-Saclay, 91401 Orsay Cedex, France

## Abstract

**Motivation:**

FIB-SEM (Focused Ion Beam—Scanning Electron Microscopy) is a technique to generate 3D images of samples up to several microns in depth. The principle is based on the alternate use of SEM to image the surface of the sample (a few nanometers thickness) and of FIB to mill the surface of the sample a few nanometers at the time. In this way, huge stacks of images can thus be acquired.

Although this technique has proven useful in imaging biological systems, the presence of some visual artifacts (stripes due to sample milling, detector saturation, charge effects, focus or sample drift, etc.) still raises some challenges for image interpretation and analyses.

**Results:**

With the aim of meeting these challenges, we developed a freeware (SEM3De) that either corrects artifacts with state-of-the-art approaches or, when artifacts are impossible to correct, enables the replacement of artifactual slices by an in-painted image created from adjacent non-artifactual slices. Thus, SEM3De improves the overall usability of FIB-SEM acquisitions.

**Availability and implementation:**

SEM3De can be downloaded from https://sourceforge.net/projects/sem3de/ as a plugin for ImageJ.

## 1 Introduction

FIB-SEM (Focused Ion Beam—Scanning Electron Microscopy) is a technique to generate 3D images of samples up to several microns in depth. It bridges the gap between serial block face SEM and transmission electron tomography in terms of field of view and resolution (Xu and Hess 2011, Peddie and Collinson 2014). The principle is based on the alternate use of SEM to image the surface of sample (a few nanometers thickness) and of FIB to mill the surface of the sample a few nanometers at the time. Alternating imaging and milling allow acquiring large images stacks.

Although this technique has proven useful in imaging biological systems (Kizilyaprak *et al.* 2014, Xu *et al.* 2021), it still presents some challenges that may limit its broader use in biological sciences. Among the most common challenges, it is worthwhile to mention image anisotropy, presence of some artifacts (presence of stripes, charge effects, focus or sample drift, and detector saturation), and time required to acquire stacks of images. In recent years, the data anisotropy problem has been successfully addressed by interpolation using deep learning (Heinrich *et al.* 2017) or optical flow (González-Ruiz et al. 2022) algorithms. Likewise, stripes can be removed by compressed sensing approaches (Schwartz *et al.* 2019) and charge effects can be addressed by using “rolling-ball”-based algorithms as implemented in ImageJ (Sternberg 1983). Focus correction is done by deconvolution-based approach as described by Fernandez *et al.* (2020). Finally, sample drift can be rectified by multi-scale cross-correlation (Messaoudi *et al.* 2013). However, there are times when artifacts are too prominent to be corrected (or simply cannot be addressed as for detector saturation). This, in turn, result in images that are too degraded to be used in quantitative analyses. In these cases, and especially if the degraded image is one occurring around the middle of the stacks (i.e. capturing the middle portion of the sample’s volume), the entire dataset is most often discarded. The only way to salvage an image stack with missing or degraded frames is to proceed with image restoration. Approaches addressing the problem of image anisotropy could theoretically be used to handle the problem of image restoration. However, there is also a number of inpainting methods designed for this very purpose (Bertalmío *et al.* 2014).

We developed a freeware (SEM3De) that improves usability of FIB-SEM acquisitions by correcting the above-mentioned artifacts with state-of-the-art approaches and, when correction is not possible, removing artifactual images and replacing them by new inpainted ones. Here, from the several existing inpainting methods, we describe the use of those based on continuity of object contours.

## 2 Methods

### 2.1 Inpainting approaches in SEM3De

Inpainting is a large family of algorithms dedicated to the restoration of missing image parts. The different kind of algorithms may be classified into two main families: textural and structural inpainting. Textural inpainting includes patch-based (Demir and Ünal 2018) or exemplar-based (Buyssens *et al.* 2015) inpainting. These algorithms try to find a texture that fits correctly in the missing area by using an “atlas of textures,” either created from the image itself or from a catalog of reference images. This family of approaches is not adapted for FIB-SEM images because it can generate non-existing objects by duplicating entire texture patches in images. The family of structural inpainting approaches (Bertalmío *et al.* 2014, Schönlieb 2015) works by extending contours to get the structures of objects back. In this family, algorithms are based on partial differential equation resolution, on total variation minimization or isophote propagation. The restoration is propagated using information from nearest pixels/voxels to the missing ones. This reduces the risk of generating non-existing objects. The drawback of these approaches is that large areas cannot be restored and, if an object fits entirely in the missing region, it cannot be recovered. Therefore, structural inpainting is suitable when few regions are missing or degraded (Masnou 2002), for instance when only a few images need to be restored in a FIB-SEM stacks.

Four algorithms are proposed in SEM3De. The first one is a simple multiscale processing (Adelson *et al.* 1984) close to linear interpolation of pixels. Images are scaled down until no pixel is missing and values are propagated from scaled-down images. The second algorithm iteratively uses discrete cosine transform to smooth evenly spaced data (Garcia 2010) and thus to reconstruct missing data in-between images in a stack. This is the default inpainting method in Matlab, we recoded it in java for use in ImageJ. The third one is Harmonic inpainting (Schönlieb 2015). The algorithm uses partial differential equations to solve the problem of missing data. It diffuses information homogeneously from known areas in all directions using the Laplacian operator. Finally, the fourth algorithm uses the principle of compressed sensing. It consists of iteratively filtering data, with a total variation minimization, while reinjecting original pixels values where they were defined (Schönlieb 2015).

### 2.2 SEM3De implemented artifacts corrections

SEM3De proposes the following approaches to artifacts correction:

1. Charge effects are visible as gradients or “ghosting” effects on images. Most of them can be removed using a classic rolling ball algorithm (Sternberg 1983).
2. Stripe removal from images, the corresponding frequencies in the Fourier space representation of images can be removed and images reconstructed. Some additional artifacts can arise due to the missing information as the suppressed frequencies contain information on both objects and stripes. The main difficulty is to determine the best fitting-stripes form (removing enough frequencies but preserving signal). Schwartz (Schwartz et al. 2019) proposed a solution by retrieving the missing data via compressed sensing using total variation minimization. This approach by Schwartz was implemented and is available in the software.
3. The best results with the FIB-SEM technique require that the focus is maintain on the top of the sample throughout data acquisition. However, as the sample is milled in between image acquisi-tions, there can be a focus drift from one acquisition to the other. Fernandez (Fernandez et al. 2020) proposed to determine the point spread function of each image and use it in a deconvolution algorithm to improve image quality and correct for focus drift. SEM3De implements this approach of Point Spread Function (PSF) determination and improves the algorithm by offering the possibility to choose the deconvolution algorithm from those available in DeconvolutionLab2 (Sage et al. 2017).
4. Sample drift is corrected using cross-correlation approach in a multi-scale optimization.

## 3 Results

**Figure 1.**
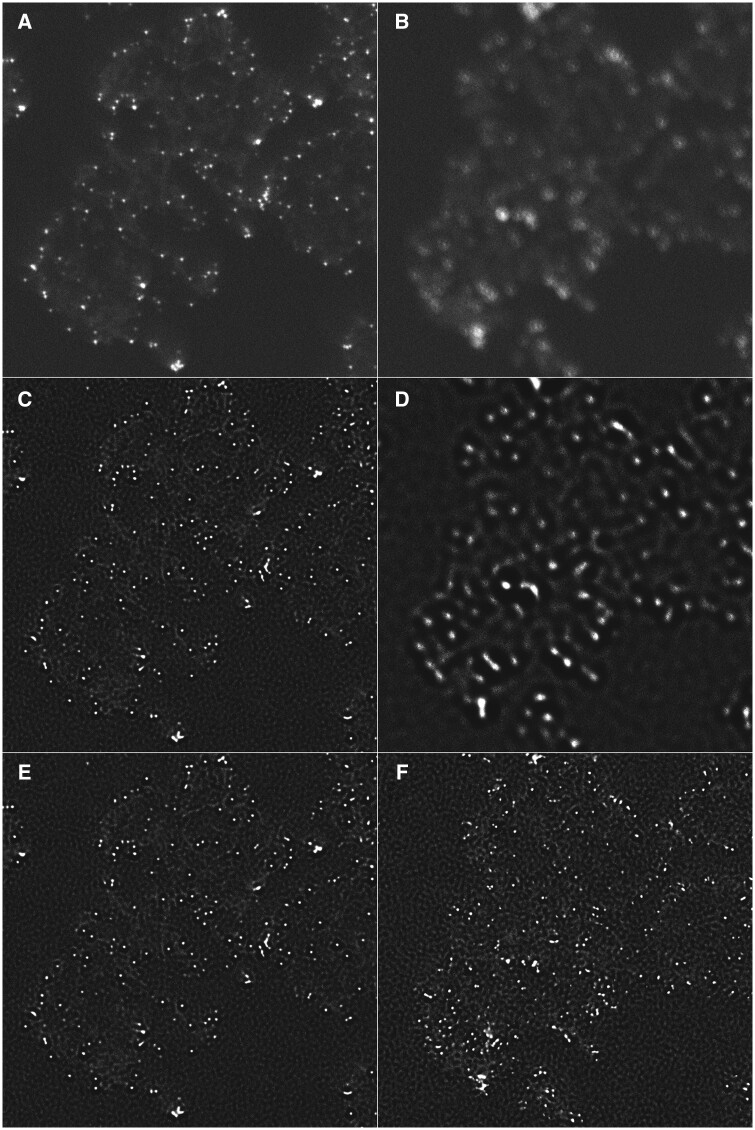
Example of use of inpainting to restore data using different implemented algorithms. Two slices from a FIB-SEM volume are shown in A (no-artifactual, slice 283) and B (artifactual, slice 293). The slice in B is strongly out of focus. C and D are the slices corresponding to A and B after the deconvolution step. The gain in the spot’s definition is visible in C but for D the defocus was too strong to be compensated. E and F (corrected) are the slices corresponding to C and D after removal and inpainting of slice D. E was not changed compared to C. F shows an image with same definition as E and in accordance with densities visible in D.

SEM3De was tested on gold-labeled tetanus antigen adsorbed into AlOOH adjuvants. During acquisition of data on this sample, focus variation and sample drift occurred making the images stack useless for further analyses. Two slices are shown in Fig. 1, the first (slice 283 shown in A) corresponds to an acquisition without artifacts and the second (slice 293 shown in B) to an artifactual one.

The first step to improve the artifactual image was to apply a charge effect correction, followed by focus correction using Richardson–Lucy algorithm with 50 iterations. This algorithm was used because it was the one giving the smaller area for gold beads. The version without total variation regularization was preferred for speed computation. The resulting images are displayed in Fig. 1C and D.

To evaluate the sharpness of images, we used the standard deviation of filtered images using a Laplacian of Gaussian. The gain is visible on slice 283 and validated by the measure of sharpness increasing from 966 to 3271. On the out of focus image (plane 293), the same filtering also improves the image’s sharpness from 826 to 1125. However, the resulting image (Fig. 1D) is not quite satisfactory and does not allow further analysis due to artifactually generated low frequencies. In this case, such as for out of focus images 225, 237, 294, and 295, also selected on sharpness measurement; these images were removed and inpainted using DCT (Garcia 2010). This inpainting approach increased values close to non-artifactual images, i.e. 3540 for slice 283 (Supplementary Fig. S1).

To estimate the acceptability of the solution provided by inpainting we have chosen a numerical quality descriptor able to consider simultaneously all structures occurring in the images independently of their sizes shapes and potential imaging artifacts. A descriptor fitting these criteria is the Fourier Ring Correlation (FRC), which measures the signal coherence between the Fourier transforms of 2 images. The FRC is defined as:

$$\mathrm{FRC}\left(r\right)=\;\frac{\sum_{r_{i}\in r}F_{1}\left(r_{i}\right)\cdot F_{2}{\left(r_{i}\right)}^{*}}{\sqrt{\sum_{r_{i}\in r}{\left|F_{1}\left(r_{i}\right)\right|}^{2}\cdot \sum_{r_{i}\in r}{\left|F_{2}\left(r_{i}\right)\right|}^{2}}}.$$

Where $F_{1}$ is the Fourier transform of image 1, ${F_{2}}^{*}$ is the complex conjugate Fourier transform of image 2, and $r_{i}$ is the individual pixel element at radius r. We used the global FRC score computed as the average of all FRC(*r*).

The comparison was done between no artifactual images and corrected ones with a range of 3 planes (Fig. 1F). The range of 3 planes was chosen because 3 consecutive slices are artifactual on this experimental dataset. Thus, the comparison for artifactual images is done with images that are not directly next to any artifactual image. We can observe that before inpainting the correlation score is lower for out of focus images than for focused images. After inpainting the average global FRC score increase to a better value than the one obtained for non-artifactual images. However, the created data are plausible since no significant differences on global-FRC are observed between non-artifactual and corrected images (Supplementary Fig. S2), whereas both significatively differs from the artifactual ones. Therefore, corrected data can now be used for further (quantitative) analysis.

To evaluate accuracy of SEM3DE, two additional public datasets (Lucchi *et al.* 2013, Ludin *et al.* 2016) have been tested. The test was performed by removing images from the full datasets and comparing the restored sets to the original ones (Supplementary S3 and S4).

## 4 Conclusion

SEM3De offers a toolbox to improve the quality of FIB-SEM stacks by correcting several common artifacts. It also integrates a number of inpainting solutions to address situations where standard artifact correction approaches are not sufficient. In these cases, artifactual images can be removed from the stacks and replaced with inpainted ones. Process has been parallelized improving proceeding time up to 8 (Supplementary S5).

SEM3De can be downloaded from https://sourceforge.net/projects/sem3de/ as a plugin for ImageJ (screenshots in Supplementary S6–S9).

## Supplementary Material

vbad119_Supplementary_Data

## Data Availability

Data corresponding to gold-labeled tetanus antigen adsorbed into AlOOH adjuvants can not be shared due to intellectual property rights. The data underlying this article supplementary were derived from sources in the public domain: EMPIAR, https://www.ebi.ac.uk/empiar/EMPIAR-10310; EPFL, https://www.epfl.ch/labs/cvlab/data/data-em. Software is publicly available on sourceforge https://sourceforge.net/projects/sem3de/.
