# Supplementary material for: SEM3De: image restoration for FIB-SEM
Source: Bioinform Adv. 2023 Sep 6;3(1):vbad119. doi: 10.1093/bioadv/vbad119 (PMC10516526; doi:10.1093/bioadv/vbad119)
Supplement: vbad119_Supplementary_Data [file vbad119_supplementary_data.docx]

**Supplementary**

Figure S1: Sharpness measurement on slices before and after inpainting


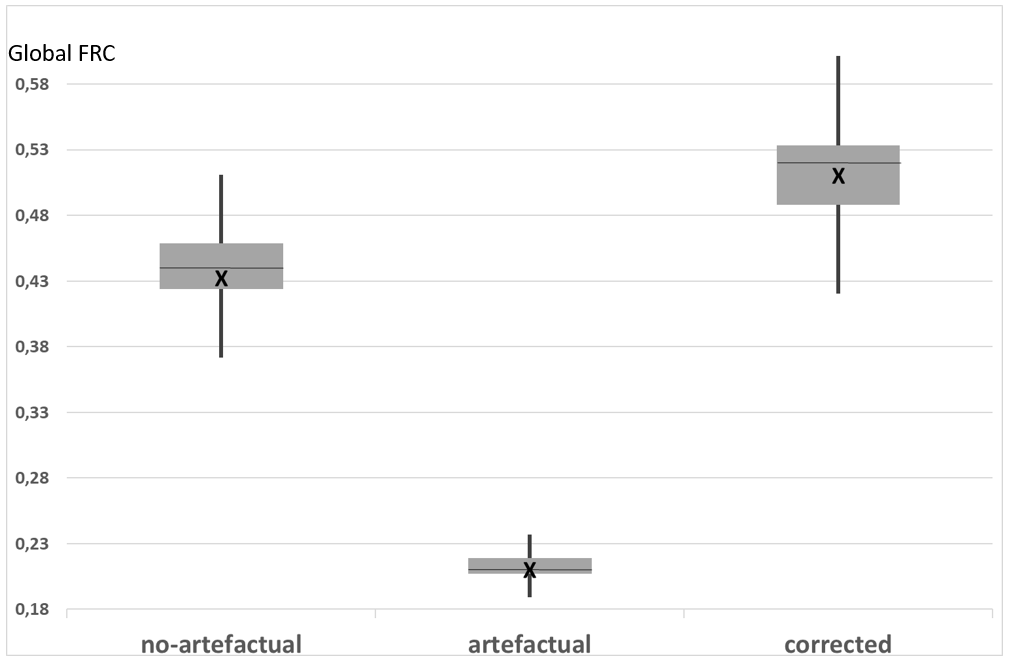


Figure S2: Evaluation of inpainting based on global FRC. Fourier correlation between slices separated by three. No-artefactual corresponds to slices that are at focus (detected using sharpness). Artefactual corresponds to slices out-of-focus correlated with images at focus. Corrected corresponds to same slices as artefactual after removal and inpainting. Horizontal line in box correspond to median. X represents average. The boxes correspond to interquartile range. Vertical lines correspond to the interval between upper and lower outer fences. Global FRC defined as the area Under the FSC curve between two samples.


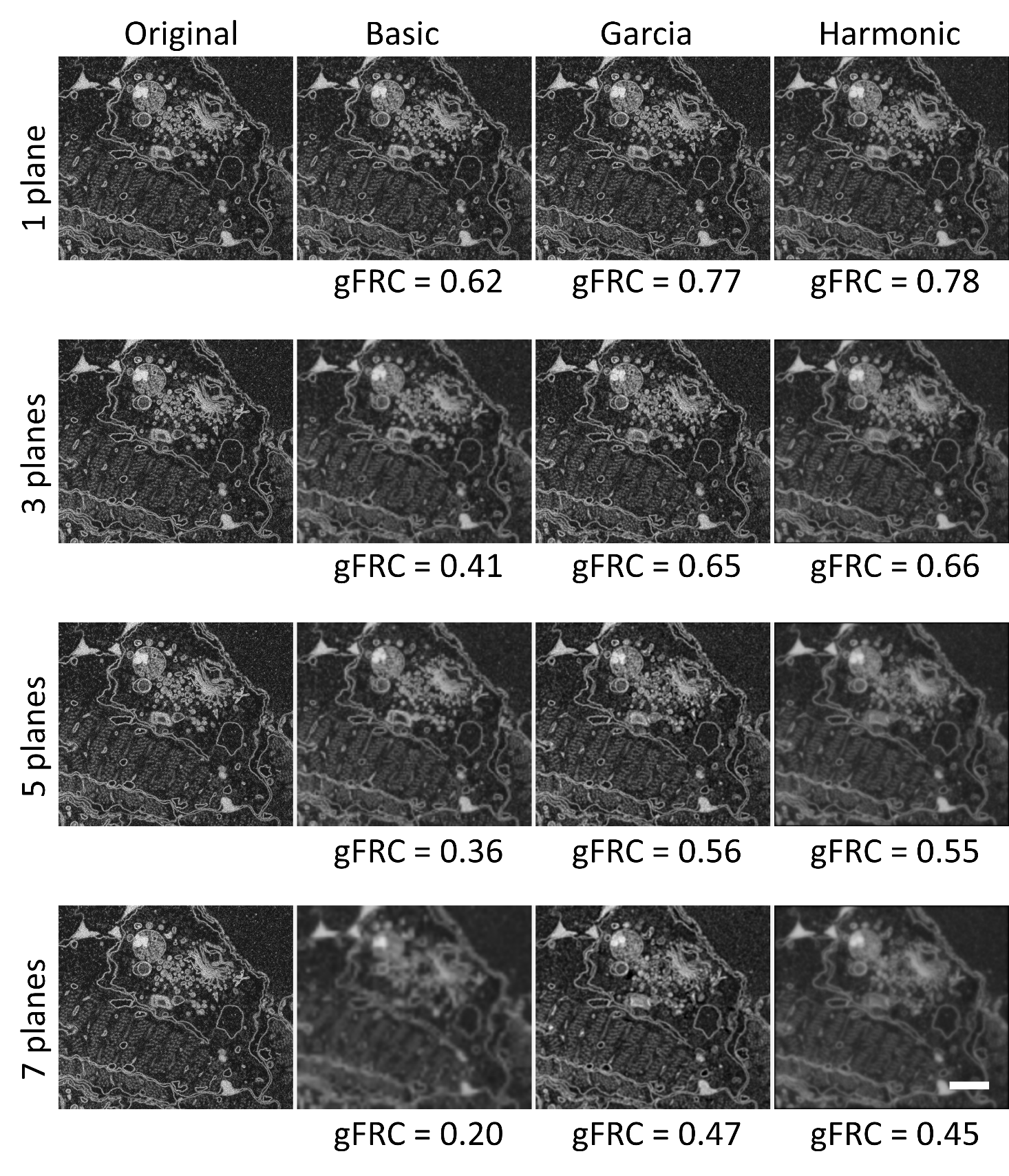


Figure S3 comparison of inpainting methods on EMPIAR 10310 data. The dataset is deposited at the Electron Microscopy Public Image Archive (EMPIAR) (ludin et al., 2016) and accessible with ID 10310 (<https://www.ebi.ac.uk/empiar/EMPIAR-10310>). A cropped region was used for the testing. 1, 3, 5 or 7 planes were removed (rows) and then inpainting methods were applied to restore them. The central plane of the inpainted planes was compared to the original plane using global FRC. The first column corresponds to original central plane, the second corresponds to basic inpainting, the third to Garcia algorithm and the fourth to Harmonic inpainting. The global FRC score is displayed bellow each image. Scale bar corresponds to 1 µm.


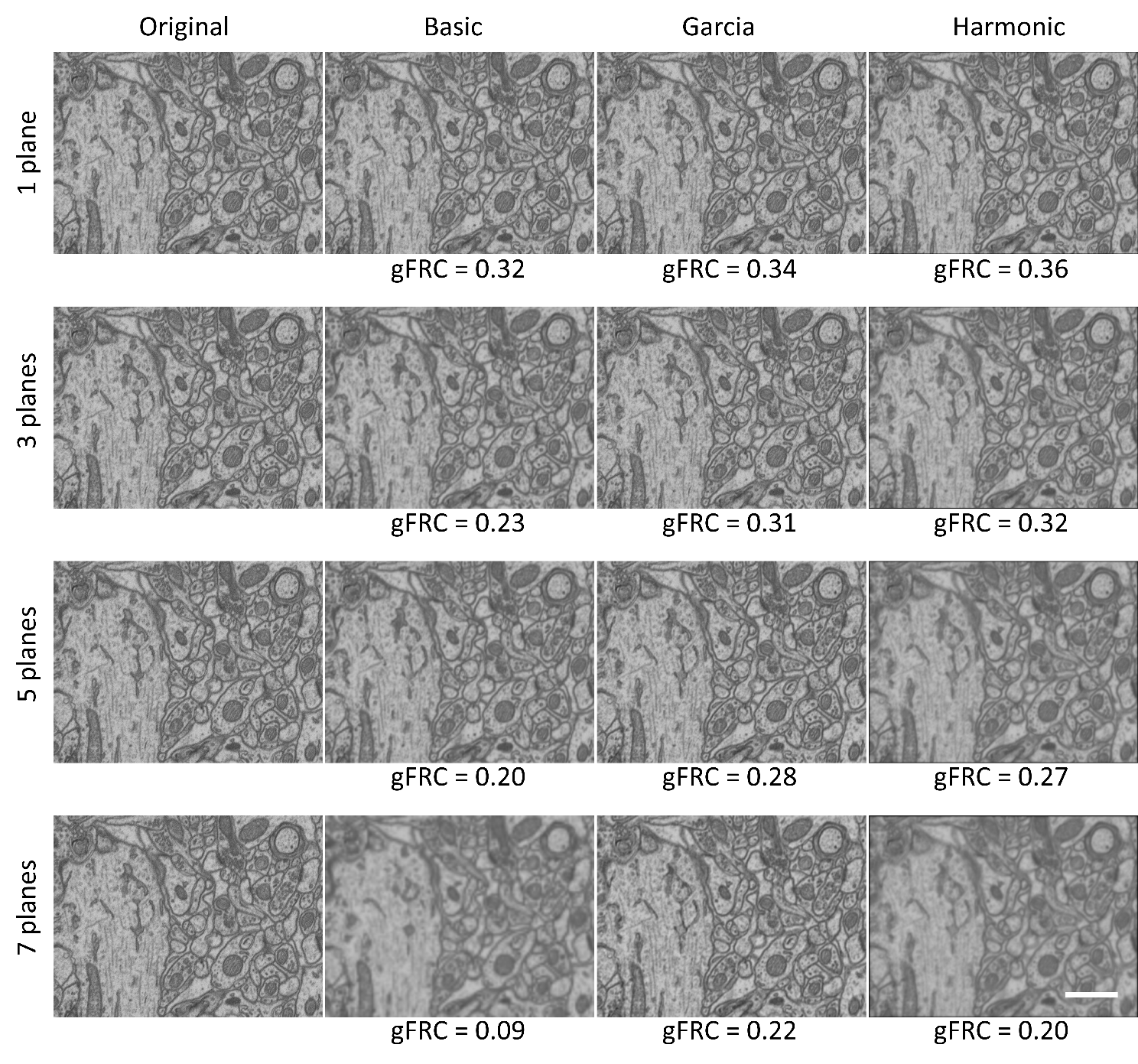


Figure S4 comparison of inpainting methods on Lucchi dataset

This dataset is publicly available (<https://www.epfl.ch/labs/cvlab/data/data-em> ) and is often used to assess automated segmentation methods (Lucchi et al., 2013). A cropped region was used for the testing. 1, 3, 5 or 7 planes were removed (rows) and then inpainting methods were applied to restore them. The central plane of the inpainted planes was compared to the original plane using global FRC. The first column corresponds to original central plane, the second corresponds to basic inpainting, the third to Garcia algorithm and the fourth to Harmonic inpainting. The global FRC score is displayed bellow each image. Scale bar corresponds to 1 µm.

-
- Figure S5 multithread performance of inpainting by the method Garcia.
- The computations were performed on a dell Precision 7920 Workstation with 2 x Intel Xeon 5220R at 2.2 GHZ (total 48 cores and HT disabled) and 64 GB of RAM. The thread limitation was defined in ImageJ and used by the software to limit the number of threads processed in parallel
- Interface
-
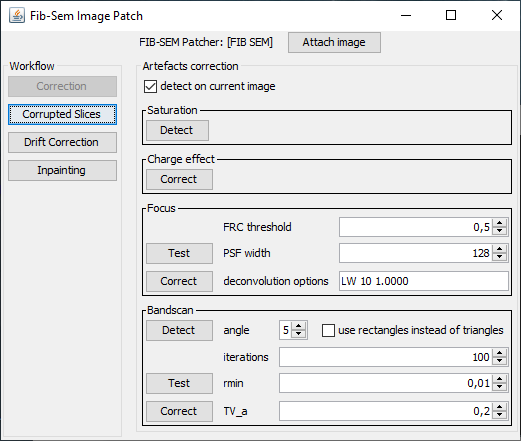

- Figure S6 software interface for the correction of artifacts.
-
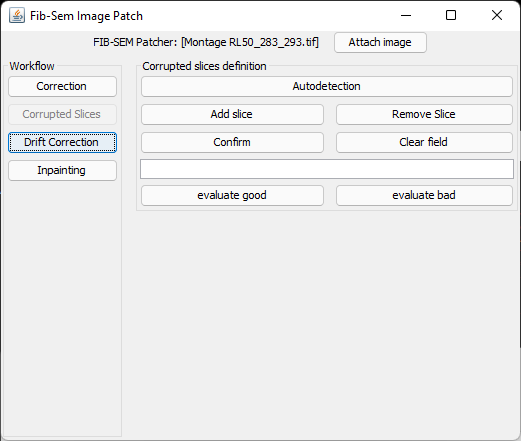

- Figure S7 software interface for the definition of artefactual images.
-
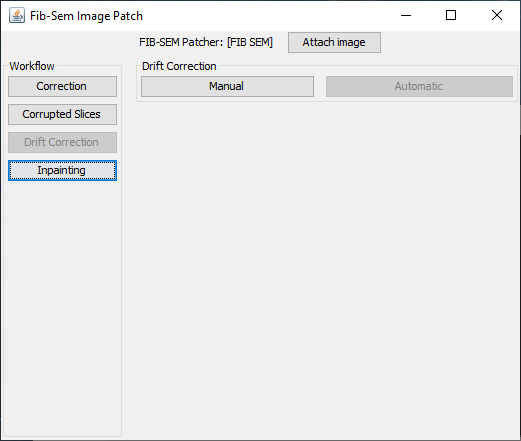

- Figure S8 software interface for the correction of drifts.
-
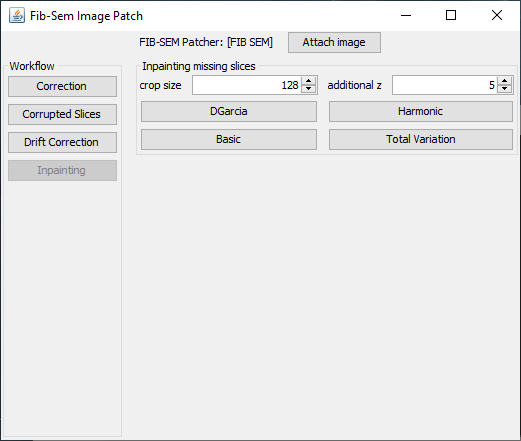

- Figure S9 software interface for the inpainting.
